# Supplementary material for: Short- and Long-Term Mortality Trends in STEMI-Cardiogenic Shock over Three Decades (1989–2018): The Ruti-STEMI-Shock Registry
Source: J Clin Med. 2020 Jul 27;9(8):2398. doi: 10.3390/jcm9082398 (PMC7465647; doi:10.3390/jcm9082398)
Supplement: Supplementary file 1 [file jcm-09-02398-s001.pdf]

Suppl Table 1 ICCU and in-hospital complications of STEMI-CS patients across the six study periods

	Period 1<br>1989-1993<br>(N = 80)	Period 2<br>1994-1998<br>(N = 68)	Period 3<br>1999-2003<br>(N = 49)	Period 4<br>2004-2008<br>(N = 91)	Period 5<br>2009-2013<br>(N = 102)	Period 6<br>2014-2018<br>(N = 103)	P for trend
**Reinfarction, %**	7.5	4.2	6.1	9.9	3.9	1.9	0.036
**Primary VF, %**	31.3	25.0	22.4	23.1	16.7	32.0	0.132
**VT, %**	26.3	19.1	18.4	31.9	22.5	19.4	0.273
**AV block, %**	52.6	45.5	44.0	40.0	30.0	29.5	0.208
**AFib/Flutter, %**	18.8	19.1	26.5	29.7	27.5	20.4	0.015
**VS Rupture, %**	6.3	7.4	8.2	6.6	8.8	5.8	0.968
**PM Rupture, %**	1.3	4.4	2.0	5.5	3.9	1.9	0.592
**FW Rupture, %**	3.8	11.8	6.1	5.5	6.9	5.8	0.498
**RV dysfunction, %**	62.3	57.6	48.0	46.7	46.0	45.5	0.511
**ICCU LoS, days**	5.3	3.9	6.6	6.5	5.7	5.7	0.137

VF, Ventricular Fibrillation; VT, Sustained ventricular Tachycardia; AV, Atrioventricular; AFib, atrial fibrillation; VS, Ventricular septum; PM, Papillary muscle; RV, Right ventricle; ICCU, Intensive Cardiovascular Care Unit; LoS, length of Stay.
